# 2635. Introduction of point-of-care testing for respiratory viruses in congregate living facilities

**DOI:** 10.1093/ofid/ofad500.2247

**Published:** 2023-11-27

**Authors:** Charlie Tan, Marianna Ofner, Jacyln O’Brien, Christina Chan, Robert Kozak, Neethu Thomas, Heather Candon, Adrienne Chan, Jerome A Leis

**Affiliations:** University of Toronto, Toronto, Ontario, Canada; Sunnybrook Health Sciences Centre, Toronto, Ontario, Canada; Sunnybrook Health Sciences Centre, Toronto, Ontario, Canada; Sunnybrook Health Sciences Centre, Toronto, Ontario, Canada; Sunnybrook Health Sciences Centre, Toronto, Ontario, Canada; Sunnybrook Health Sciences Centre, Toronto, Ontario, Canada; Sunnybrook Health Sciences Centre, Toronto, Ontario, Canada; Sunnybrook Health Sciences Centre, Toronto, Ontario, Canada; Sunnybrook Health Sciences Centre, Toronto, Ontario, Canada

## Abstract

**Background:**

Timely diagnosis of viral respiratory infection is critical to control respiratory viral transmission in congregate care facilities. We conducted a quality improvement study to assess whether a point-of-care (POC) testing for detection of respiratory viruses could improve time to outbreak measures in congregate living facilities.

**Methods:**

We implemented a POC cartridge based nucleic acid amplification test platform, which detects SARS-CoV-2, influenza A, and respiratory syncytial virus, at one long-term care home and three retirement homes between December 1, 2022 and April 15, 2023. Residents with respiratory symptoms underwent paired testing, whereby nasopharyngeal swabs were tested first on POC, then on multiplex respiratory virus panel (MRVP) polymerase chain reaction at an external laboratory. We determined difference in turn-around time (TAT), as well as sensitivity, specificity, positive predictive value (PPV) and negative predictive value (NPV), using MRVP as the reference standard. We also compared time to outbreak declaration (from MRVP result) to five other congregate care facilities without access to POC testing.

**Results:**

A total of 189 tests were performed using POC testing, of which 39.7% (75/189) were positive. SARS-CoV-2 was the most common virus identified (90.7%, 68/75). Median difference in TAT between POC and MRVP was 34.0 hours (IQR 21.8–43.5 hours). Sensitivity, specificity, PPV and NPV of POC were 0.88, 0.87, 0.83 and 0.90, respectively. There were six outbreaks during the study period (all SARS-CoV-2), of which two were declared based on POC result alone, before MRVP was resulted. Median time to outbreak declaration from MRVP result for the remaining four outbreaks was 6.6 hours (IQR 4.6–9.2 hours). In comparison, there were nine outbreaks at the non-POC comparator facilities; median time to outbreak declaration was 15.2 hours (IQR 5.5–26.0 hours).

**Conclusion:**

We demonstrated the feasibility of implementing a POC platform in congregate living facilities for identification of respiratory viral infection. The improved TAT resulted in more expedited detection and declaration of outbreaks.

**Disclosures:**

**Jerome A. Leis, MD MSc FRCPC**, Ontario Hospital Association, Ministry of Attorney General of Ontario, Seneca College: Expert Testimony

